# Acidosis Is a key regulator of osteoblast ecto‐nucleotidase pyrophosphatase/phosphodiesterase 1 (NPP1) expression and activity

**DOI:** 10.1002/jcp.25041

**Published:** 2015-08-24

**Authors:** Isabel R. Orriss, Michelle L. Key, Mark O.R. Hajjawi, José L. Millán, Timothy R. Arnett

**Affiliations:** ^1^ Department of Comparative Biomedical SciencesRoyal Veterinary CollegeLondonUK; ^2^ Department of Cell and Developmental BiologyUniversity College LondonLondonUK; ^3^ Sanford‐Burnham Medical Research InstituteLa JollaCalifornia

## Abstract

Previous work has shown that acidosis prevents bone nodule formation by osteoblasts in vitro by inhibiting mineralisation of the collagenous matrix. The ratio of phosphate (P_i_) to pyrophosphate (PP_i_) in the bone microenvironment is a fundamental regulator of bone mineralisation. Both P_i_ and PP_i_, a potent inhibitor of mineralisation, are generated from extracellular nucleotides by the actions of ecto‐nucleotidases. This study investigated the expression and activity of ecto‐nucleotidases by osteoblasts under normal and acid conditions. We found that osteoblasts express mRNA for a number of ecto‐nucleotidases including NTPdase 1–6 (ecto‐nucleoside triphosphate diphosphohydrolase) and NPP1‐3 (ecto‐nucleotide pyrophosphatase/phosphodiesterase). The rank order of mRNA expression in differentiating rat osteoblasts (day 7) was *Enpp1* > *NTPdase 4* > *NTPdase 6 *> *NTPdase 5* > alkaline phosphatase > ecto‐5‐nucleotidase > *Enpp3 *> *NTPdase 1* > *NTPdase 3* > *Enpp2* > *NTPdase 2*. Acidosis (pH 6.9) upregulated NPP1 mRNA (2.8‐fold) and protein expression at all stages of osteoblast differentiation compared to physiological pH (pH 7.4); expression of other ecto‐nucleotidases was unaffected. Furthermore, total NPP activity was increased up to 53% in osteoblasts cultured in acid conditions (*P* < 0.001). Release of ATP, one of the key substrates for NPP1, from osteoblasts, was unaffected by acidosis. Further studies showed that mineralised bone formation by osteoblasts cultured from NPP1 knockout mice was increased compared with wildtypes (2.5‐fold, *P* < 0.001) and was partially resistant to the inhibitory effect of acidosis. These results indicate that increased NPP1 expression and activity might contribute to the decreased mineralisation observed when osteoblasts are exposed to acid conditions. J. Cell. Physiol. 230: 3049–3056, 2015. © 2015 The Authors. *Journal of Cellular Physiology* Published by Wiley Periodicals, Inc.

**Abbreviations**
ANK: progressive ankylosis protein
ATP: adenosine triphosphate
LDH: lactate dehydrogenase
NDP: nucleotide diphosphate
NMP: nucleotide monophosphate
NPP: ecto nucleotide pyrophosphatase/phosphodiesterase
NTP: nucleotide triphosphate
NTPdase: ecto nucleoside triphosphate diphosphohydrolase
P_i_: phosphate
PP: pyrophosphate
TNAP: alkaline phosphatase
Ttw/Ttw: tip toe walking
UTP: uridine triphosphate

The negative actions of acidosis on the skeleton have long been known (Jaffe et al., 1932). Bone functions as a ‘fail‐safe’ store of alkali available to buffer metabolic acid if systemic pH falls below the normal, narrow limits (Arnett, 2010). The solubility of bone mineral is highly pH‐sensitive, increasing sharply when pH is decreased below 7.0 (Neuman and Neuman, 1958).

Acidosis also has direct effects on bone cells. Small pH drops within the pathophysiolocial range result in large increases in the resorptive activity of osteoclasts in vitro (Arnett and Dempster, 1986). Acidosis (pH 6.9) also prevents the formation of mineralised bone nodules in primary cultures of osteoblasts. Part of this inhibition can be attributed to physico‐chemical dissolution of hydroxyapatite (Brandao‐Burch et al., 2005); however, expression and activity of tissue non‐specific alkaline phosphatase (TNAP) by osteoblasts is also strikingly decreased in acid conditions, suggesting an additional cell‐mediated component (Krieger et al., 1992; Brandao‐Burch et al., 2005). Collagen formation and deposition are unchanged at pH6.9 suggesting that the effects of acidosis are restricted to the process of mineralisation (Brandao‐Burch et al., 2005).

Bone mineralisation depends on a constant supply of Ca^2+^ and phosphate (P_i_) ions for hydroxyapatite crystal formation. Pyrophosphate (PP_i_) is a ubiquitous, potent inhibitor of mineralisation (Fleisch and Bisaz, 1962), and mineralisation in the bone microenvironment ultimately depends on the ratio of P_i_ to PP_i_ concentrations (Felix and Fleisch, 1976; Kornak, 2011). Both P_i_ and PP_i_ can be generated from extracellular nucleotide triphosphates (NTPs) such as ATP and UTP by the actions of ecto‐nucleotide pyrophosphatase/phosphodiesterases (NPPs), ecto‐nucleoside triphosphate diphosphohydrolases (NTPdases), ecto‐5‐nucleotidase and TNAP (Stefan et al., 2005; Orriss et al., 2007). Many ecto‐nucleotidases have overlapping specificities. For example, NTPdases catalyse the reactions: NTP → nucleotide diphosphate (NDP) + P_i_ and NDP → nucleotide monophosphate (NMP) + P_i_, whereas NPPs primarily hydrolyse NTP → NMP + PP_i_ or NDP → NMP + P_i_ (Zimmermann et al., 2012). Osteoblasts have been shown to express three members of the NPP family (NPP1,2,3) (Johnson et al., 2000; Hessle et al., 2002; Orriss et al., 2007). TNAP is the main enzyme involved in PP_i_ breakdown and the key source of P_i_, whilst NPP1 is thought to be most important in PP_i_ generation (Hessle et al., 2002). Thus, the opposing actions of TNAP and NPP1 are critical in determining the extracellular P_i_/PP_i_ ratio and, therefore, the level of skeletal mineralisation (Hessle et al., 2002; Johnson et al., 2000).

The important role of NPP1 in bone mineralisation is highlighted by several different knockout models; the naturally occurring NPP1 ‘knockout’ termed the tip‐toe walking (*ttw/ttw)* mouse and the genetically altered NPP1 knockout (*Enpp1^−/−^*). The *ttw/ttw* mouse displays ossification of the spinal ligaments, peripheral joint hyperstosis and calcification of articular cartilage (Okawa et al., 1998). The phenotype of *ttw/ttw* mice also has similarities to the human disease ‘Ossification of the posterior longitudinal ligament of the spine’ (OPLL). The *Enpp1^−/−^* model displays a number of defects related to impaired PP_i_ production including calcification of the aorta, spine, joints, cartilage and whisker vibrissae and increased mineralisation of the osteocyte lacunae (Johnson et al., 2003; Harmey et al., 2004; Mackenzie et al., 2012b; Hajjawi et al., 2014). Surprisingly, given the reduction in extracellular PP_i_ levels, *Enpp1^−/−^* mice display reduced trabecular and cortical bone in the long bones and decreased bone strength (Mackenzie et al., 2012b). Recently, an alternative knockout model has also been reported (*Enpp1^asj^*) (Li et al., 2013); these animals, which are on a different genetic background to *Enpp1^−/−^* mice, display many of the same phenotypic characteristics such as widespread ectopic calcification (Li et al., 2013).

Our previous work showed that decreased TNAP expression and activity contributes to the inhibitory effects of acidosis on bone mineralisation (Brandao‐Burch et al., 2005). Given that NPP1 is also a key regulator of mineralisation, the aim of this study was to determine whether acidosis influences the expression and activity of NPP1 and other P_i_ and PP_i_‐generating enzymes.

## Materials and Methods

### Reagents

All tissue culture reagents were purchased from Life Technologies (Paisley, UK); unless otherwise mentioned, other reagents were obtained from Sigma–Aldrich (Poole, Dorset, UK). Molecular biology reagents were purchased from Invitrogen (Paisley, UK) and Qiagen Ltd (Crawley, UK). Primers for RT‐PCR and qPCR were from MWG Biotech (Ebersberg, Germany) and Qiagen, respectively. The NPP1 antibody was obtained from Thermo Fisher Scientific (Loughborough, UK) and the β‐actin antibody from Abcam (Cambridge, UK).

### Osteoblast cell culture

Primary rat osteoblast cells were obtained from 2‐day‐old neonatal Sprague–Dawley rats as described previously (Orriss et al., 2012b; Taylor et al., 2014). All animal experiments were approved by the University College London Animal Users Committee and the animals were maintained in accordance with the UK Home Office guidelines for the care and use of laboratory animals.

Osteoblasts were cultured at pH 7.4 or 6.9 for up to 14 days, with half medium changes every 2–3 days. Experiments were carried out at 3–4 time points during the osteoblast culture; day 4 (proliferating pre‐osteoblasts), day 7 (differentiating osteoblasts), day 10 (mature osteoblasts at the onset of mineralisation) and day 14 (mature, actively mineralising osteoblasts). The pH, pO_2_ and pCO_2_ were carefully monitored throughout using a blood gas analyser (ABL705, Radiometer, Crawley, UK). Bone nodule formation by osteoblasts cultured was measured as described previously (Hoebertz et al., 2002; Orriss et al., 2012b; Taylor et al., 2014).

### Osteoblast cell culture from *Enpp1^−/−^* mice

The generation and characterisation of *Enpp1^−/−^* mice, which are on a 129Sv/TerJ genetic background, have been previously described (Sali et al., 1999). Osteoblasts were isolated from 2‐3‐day‐old wildtype (*Enpp1^+/+^*) or homozygote (Enpp1^−/−^) mice using a 4‐step process as previously described (10 mg/ml collagenase II in HBSS for 10 min, collagenase II for 30 min, 4 mM EDTA in PBS for 10 min, collagenase II for 30 min) (Orriss et al., 2014; Taylor et al., 2014). The first digest was discarded and the cells from the other three digests pooled and resuspended in α‐minimum essential medium supplemented with 10% foetal calf serum (FCS) and 5% gentimicin (complete mixture abbreviated to α‐MEM). Cells were cultured for 2–4 days in a humidified atmosphere of 5% CO_2_–95% air at 37°C in 25 cm^2^ flasks until confluent. Upon confluence, cells were sub‐cultured into 6‐well trays in α‐MEM at pH 7.4 or 6.9 and supplemented with 2 mM β‐glycerophosphate and 50 μg/ml ascorbic acid (mixture abbreviated to ‘supplemented α‐MEM’), with half medium changes every 3 days. Medium pH, pCO_2_ and pO_2_ were monitored as above.

### Total RNA extraction and complementary DNA strand synthesis

Osteoblasts were cultured at pH 7.4 or 6.9 in 6‐well trays for 7, 10 or 14 days before total RNA was extracted from three wells using TRIZOL^®^ reagent (Life Technologies, Paisley, UK) according to the manufacturer's instructions. Extracted RNA was treated with RNase‐free DNase I (35 U/ml) for 30 min at 37°C. The reaction was terminated by heat inactivation at 65°C for 10 min. Total RNA was quantified spectrophotometrically by measuring absorbance at 260 nm. For each sample, 0.5 μg of DNase‐treated total RNA was used as a template for first strand cDNA synthesis in a 20 μl reaction also containing 0.5 μg oligo dT, 3 mM MgCl_2_, 0.5 mM dNTPs, 20 U recombinant RNasin^®^ ribonuclease inhibitor, ImProm‐II^®^ 5x reaction buffer and 200 U ImProm‐II reverse transcriptase. The reaction mix was annealed for 5 min at 25°C, followed by extension at 42°C for 60 min and inactivation at 70°C for 15 min. cDNA was stored at –20°C until amplification by RT‐PCR or qPCR.

### Quantitative real time polymerase chain reaction (qRT‐PCR)

**Figure 1:**
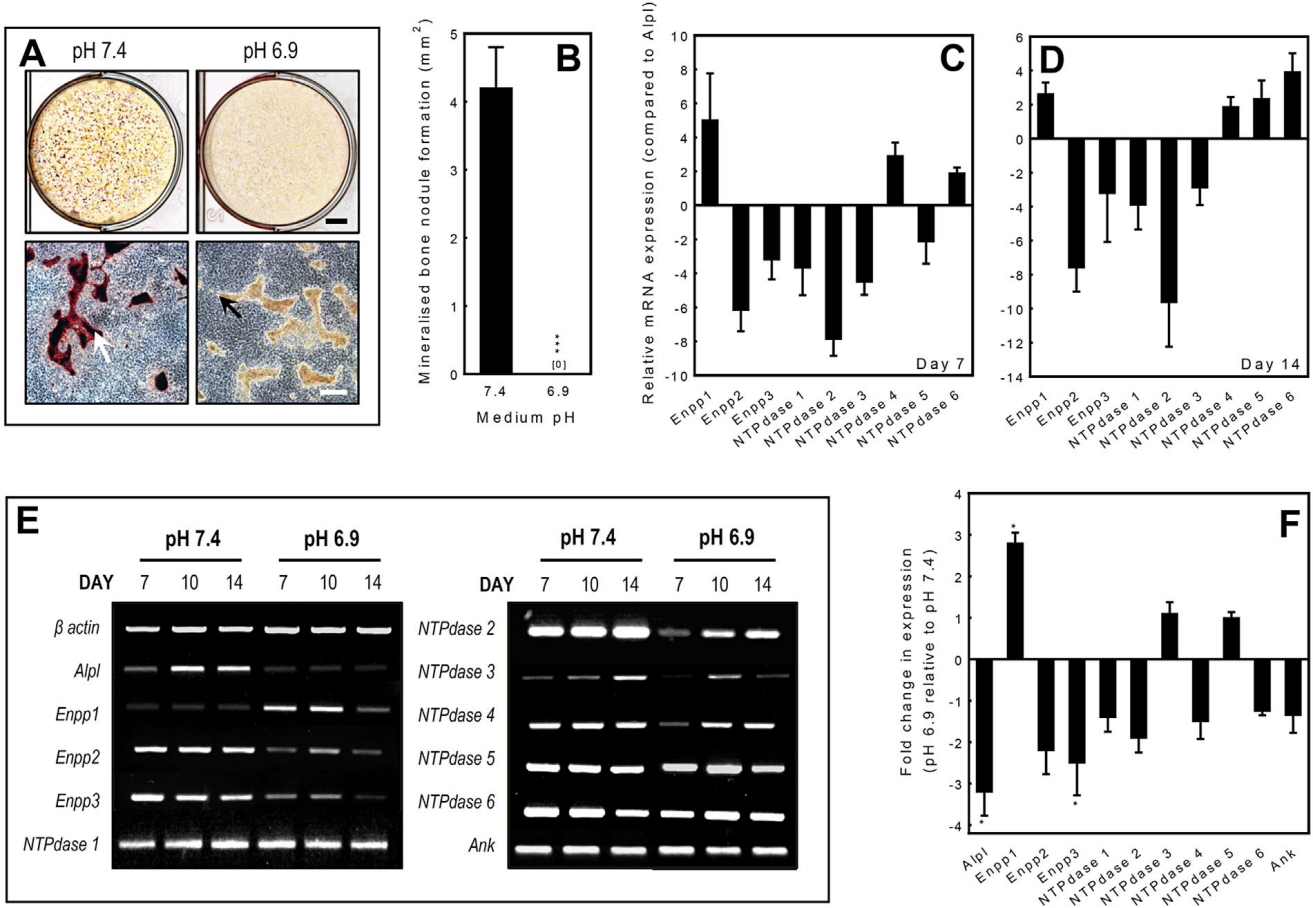
The effect of pH on mineralisation by primary rat osteoblasts and ecto‐nucleotidase mRNA expression. Osteoblasts were cultured at pH 6.9 or 7.4 for up to 14 days. A: Low power scans demonstrate a lack of alizarin red staining at pH 6.9 compared to pH 7.4. Phase contrast micrographs show the presence of collagenous matrix but the absence of alizarin red staining at pH 6.9 indicates that mineralisation has failed to occur. Scale bars: tissue culture wells = 5 mm, phase contrast micrographs = 500 μm. B: Culture at pH 6.9 results in the complete abolition of mineralised nodule formation. Values are means ± SEM (n = 6 replicate wells), ***P < 0.001. C, D: Ecto‐nucleotidase mRNA expression was investigated in differentiating (day 7) and mature, bone forming (day 14) osteoblasts at physiological pH7.4 using qRT‐PCR. Expression levels are given as relative to TNAP (*Alpl*) expression. The rank order of mRNA expression in differentiating osteoblasts was *Enpp1 > NTPdase 4 > NTPdase 6 > NTPdase 5 > Alpl > Enpp3 > NTPdase 1 > NTPdase 3 > Enpp2 > NTPdase 2*. In mature, bone forming osteoblasts the rank order of expression was *NTPdase 6 > Enpp1 > NTPdase 4 > Alpl > NTPdase 5 > Enpp3 > NTPdase 3 > Enpp2 > NTPdase 1 > NTPdase 2*. E: RT‐PCR analysis of mRNA at day 7, 10 and 14 showed that compared to pH 7.4 culture at pH 6.9 upregulated the expression of *Enpp1* mRNA in osteoblasts, at all stages of differentiation. In contrast, *Alpl*, *Enpp2*, *Enpp3* and *NTPdase 2* mRNA expression was decreased in acid. mRNA expression of *NTPdase1, 3‐6* and *Ank* was unchanged. F: qRT‐PCR analysis of osteoblasts at day 10 (the stage when the largest effects were seen with RT‐PCR) showed that culture under acid conditions resulted in a 2.8‐fold increase in *Enpp1* mRNA expression; expression of *Alpl* and *Enpp3* was decreased 3.2‐ and 2.5‐fold, respectively. The mRNA expression of the other ecto‐nucleotidases was not significantly altered. All qPCR reactions were carried out in triplicate using RNAs derived from four different osteoblast cultures. Values are means ± SEM, *P < 0.05.

Osteoblast cDNA was amplified using QuantiTect SYBR Green PCR kit (Qiagen Ltd, Crawley, UK) and specific QuantiTect primers. qRT‐PCR (Chromo4, Biorad Laboratories Ltd, Hemel Hempstead, UK) was performed according to manufacturer's instructions with 40 cycles of denaturation (95°C for 10 sec) and detection (60°C for 30 sec). Data were analysed using the Pfaffl method (Pfaffl, 2001) and is shown as the fold change in gene expression relative to TNAP (Fig. 1C–D) or cells cultured at pH 7.4 (Fig. 1F). All reactions were carried out in triplicate using RNAs derived from four different osteoblast cultures.

### Reverse transcriptase polymerase chain reaction (RT‐PCR)

Rat osteoblast derived cDNA was amplified by RT‐PCR in 25 μl reactions containing ∼0.5 μg cDNA, 0.2 mM dNTP (10 mM stock), 1.5 mM MgCl_2_, 0.2 μM of both sense and antisense primer, 1 U Taq DNA polymerase in thermophilic DNA polymerase 10x buffer. PCR was performed according to manufacturer's instructions, with cycles of denaturation at 95°C for 30 sec, annealing for 30 sec extension at 72°C for 45 sec and reaction termination at 72°C for 5 min. For analysis, PCR products were loaded on to 1% agarose gels containing 0.3 μg/ml ethidium bromide. Gels were run at ∼80 mA and the DNA position visualised by exposure to UV light. To account for differences in original cell number and cDNA quality, all samples were normalised against mRNA for β‐actin. The expression of TNAP, NPP1‐3, NTPdase 1‐6 and ANK was investigated in osteoblasts cultured for 7, 10 and 14 days. All reactions were carried out in triplicate using cDNAs derived from three different osteoblast cultures. Primer sequences and annealing temperatures are shown in Table 1.

**Table 1:** Primer sequences for RT‐PCR reactions

Gene	Primer sequence (5′–3′)	Product size (bp)	Annealing temperature (°C)
*β*‐*Actin*	S – gtt cgc cat gga tga cga t AS – tct ggg tca tct ttt cac gg	332	53
*Alpl*	S – ctc att tgt gcc aga gaa AS – gtt gta cgt ctt gga gac	238	50
*Enpp1*	S – gtc agt atg cgt gct aac AS – tgg cac act gaa ctg tag	309	49
*Enpp2*	S – gcc ctc cgt taa tca tct AS – gca gag aaa gcc act gaa	399	51
*Enpp3*	S – gca tgc aga gga att gtc AS – tgg gaa cgg tgt atg acc	396	53
*NTPdase 1*	S – ggg cct atg ggt gga tta ct AS – gta aaa gca cgg gtc ctt ga	332	58
*NTPdase 2*	S – cca gct atg caa atg aac AS – aac acc cct tca tcc tgt	256	56
*NTPdase 3*	S – cag cca aac ctt cag atg AS – tgt gcc aca ggt tct tct	356	53
*NTPdase 4*	S – agg cag ttg tgg aag tca AS – cag aaa tgg agc atc agg	362	53
*NTPdase 5*	S – tag ctt ggg tta ccg tga AS – ctc ctt cca acc atc ttg	315	53
*NTPdase 6*	S – ggg atg act gtg ttt cca AS – ttg tca tcc tca gca ggt	322	53
*ANK*	S – cat cac caa cat agc cat cg AS – aag gca gcg aga tac agg aa	320	55

### Determination of total cellular NPP activity

The NPP activity of cell lysates was determined colorimetrically (Bio‐Tek EL_X_800 plate reader, Fisher Scientific Loughborough, UK) in cells cultured at pH 7.4 or 6.9 for 7, 10 or 14 days. The assay used to measure total NPP activity was based on the method originally described by Razzell and Khorana (Razzell and Khorana, 1959). Briefly, cells were lysed in a buffer containing 1% TritonX‐100 in 0.2 M Tris base with 1.6 mM MgCl_2_, pH 8.1. Following centrifugation at 500*g*, the NPP activity of collected supernatants was measured using 5 mM p‐nitrophenyl‐thymidine 5′‐monophosphate as a substrate. Total protein in cell lysates was determined using the Bradford assay (Sigma–Aldrich, Poole, UK).

### Immunofluorescence

Rat osteoblasts were seeded onto sterile 1 cm diameter discs, cut from Melinex 75 micron clear polyester film (Du Pont, Dumfries, UK), in 24‐well trays at 2.5 × 10^4^ cells/disc, for 14 days at pH 7.4 or 6.9. Upon termination, discs were removed and fixed with 4% paraformaldehyde in 0.1 M phosphate buffer for 20 min at room temperature, washed 3 × 5 min with PBS and stored at 4°C in PBS until staining. Each disc was incubated with a blocking solution consisting of 10% FCS in PBS, for 1 h. The NPP1 antibody was diluted in blocking solution at 1:200. Discs were incubated overnight in the primary antibody solution with gentle agitation; negative controls were incubated overnight in 10% FCS in PBS, containing no antibody or antibody and blocking peptide. Cells were subjected to three 5‐min washes with PBS before incubation for 1 h with a goat anti‐rabbit Cy5‐labelled secondary antibody solution (1:400) and a DAPI counter stain (1:3,500), diluted in PBS with 1% FCS. After three further 5‐min PBS washes, discs were mounted on to microscope slides using Citifluor AF2 solution (Citifluor, London UK) and viewed by fluorescence microscopy (Cy5 absorbance and emission at 650 nm and 670 nm, respectively).

### Western blot

Osteoblasts were cultured for 4, 7, 10 or 14 days at pH 7.4 or 6.9 before the monolayers were lysed in ice‐cold radio immunoprecipitation (RIPA) lysis buffer (50 mM Tris–HCl pH 7.4, 150 mM NaCl, 5 mM EDTA, 0.1% SDS 1 mM phenyl methyl sulfonyl fluoride, 1 mg/ml aprotinin, 1 mM Na_3_VO_4_ and 2.5 mg/ml deoxicolic acid). Cell homogenates were sonicated for 5 min and stored at −80°C for at least half an hour before use. Protein concentrations from lysates were determined using the Bradford assay. Prior to loading, total protein samples were denatured by incubating at 95°C for 5 min with 5x reducing sample buffer (60 mM Tris–HCl pH 6.8, 25% glycerol, 2% SDS, 10% β‐mercaptoethanol and 0.1% bromophenol blue). Protein samples (20 μg/lane) were loaded into SDS–PAGE (8%) gels and transferred onto a polyvinylidenifluoride (PVDF) membrane (Amersham, Buckinghamshire, UK) by the use of a wet tank blotter (Bio‐Rad, Hercules, CA) at 150 V for 1 h. The membrane was afterwards blocked with 5% non‐fat milk and incubated with rabbit NPP1 (1:200) or β actin (1:2,500) antibody overnight at room temperature. After washing, blots were incubated in horseradish peroxidase‐conjugated secondary antibodies for 1 h. Immunoreactivity was visualised using Immobilon™ Western chemiluminescent HRP substrate (Millipore, Watford, UK).

### Measurement of extracellular ATP

Prior to measurement of ATP levels, culture medium was removed, cell layers washed and cells incubated with serum‐free DMEM at pH 7.4 or 6.9 (1 ml/well) for 1 h. The long‐term effects acid on ATP levels were measured in osteoblasts cultured at pH 7.4 or 6.9 for 4, 7, 10 or 14 days. All samples were immediately snap‐frozen on dry ice for later ATP quantification. ATP release was measured by luminescence using the *luciferin‐luciferase* assay as described previously (Orriss et al., 2009).

### Quinacrine staining

The acridine derivative, quinacrine, is a weak base that binds ATP with a high affinity. When excited by light at 476 nm, it emits fluorescence in the 500–540 nm range and is widely used to visualise ATP‐containing subcellular compartments in live cells. Osteoblasts were seeded onto Melinex discs, in 24 well trays at 2.5 × 10^4^ cells/disc and cultured for 7 days at pH 7.4 or 6.9. To visualise ATP filled vesicles, Melinex discs were twice washed with PBS before incubation with 30 μM quinacrine for 1 h; discs were washed twice more and mounted onto microscope slides. The cells were immediately observed using fluorescence microscopy.

### Statistical analysis

Statistical comparisons were made using both parametric (one‐way analysis of variance and adjusted using the Bonferroni method) and non‐parametric (Kruskal–Wallis and adjusted using the Dunn method) tests. In all figures where statistical significance is shown, both of these methods gave corresponding results. Unless stated, representative data are presented as means ± SEM for 6–10 replicates. Results presented are for representative experiments that were each repeated at least three times.

## Results

### Acidosis inhibits bone mineralisation of the collagenous matrix by osteoblasts

Rat osteoblasts cultured in control conditions at pH 7.4 for 14 days formed abundant mineralised bone spicules and nodules, as shown by alizarin red staining for deposited calcium. In acidotic medium (pH 6.9), mineralisation of the collagenous matrix was completely prevented as evidenced by the absence of alizarin red staining (Fig. 1A and B).

### Osteoblasts express multiple NPPs and NTPdases

Osteoblasts were cultured for up to 14 days at pH 7.4 and expression of NPPs and NTPdases was studied in differentiating osteoblasts (day 7) and mature, bone forming osteoblasts (day 14) using qRT‐PCR. Expression of mRNA was detected for TNAP (*Alpl)*, *Enpp1‐3* and *NTPdase1‐6* **(**Fig. 1C and D).

To determine which of the ecto‐nucleotidases were most highly expressed by osteoblasts, we compared the relative expressions of all the ecto‐enzymes receptors in differentiating (day 7) and mature, bone forming osteoblasts (day 14) at physiological pH 7.4. Following normalisation of expression using β‐actin, TNAP receptor expression was used as the calibrator and expression is presented as fold change relative to TNAP (*Alpl*) levels. The rank order of mRNA expression in differentiating osteoblasts was *Enpp1 > NTPdase 4 > NTPdase 6 > NTPdase 5 > Alpl > Enpp3 > NTPdase 1 > NTPdase 3 > Enpp2 > NTPdase 2* (Fig. 1C). In mature bone forming osteoblasts, the mRNA expression profile was slightly different with the rank order; *NTPdase 6 > Enpp1 > NTPdase 4 > Alpl > NTPdase 5 > Enpp3 > NTPdase 3 > Enpp2> NTPdase 1 > NTPdase 2* (Fig. 1D).

### Expression of Enpp1 mRNA is upregulated in osteoblasts cultured at pH 6.9

RT‐PCR analysis of mRNA expression at day 7, 10 and 14 demonstrated that *Enpp1* mRNA expression was upregulated in cells cultured in acidotic conditions (pH 6.9), at all stages of differentiation (Fig. 1E) compared to pH 7.4. In contrast, expression of *Alpl, Enpp2, Enpp3* and NTPdase 2 was downregulated at pH 6.9 (Fig. 1E). Expression of the other ecto‐nucleotidases and the PP_i_ transporter, *Ank*, was unaffected.

In order to quantify the effects of acidosis on ecto‐nucleotidase mRNA expression, qRT‐PCR was performed on osteoblasts at the point in culture where the largest effects were seen with RT‐PCR (day 10, mature cells at the onset of mineralisation). Compared to pH 7.4, culture at pH 6.9 increased the expression of *Enpp1* 2.8‐fold, whilst expression of *Alpl* and *Enpp3* were decreased 3.2‐ and 2.5‐fold, respectively (*P* < 0.05) (Fig. 1F). The mRNA expression of the other ecto‐nucleotidases and ANK was not significantly altered by acidosis.

### NPP1 protein expression is increased in acidosis

**Figure 2:**
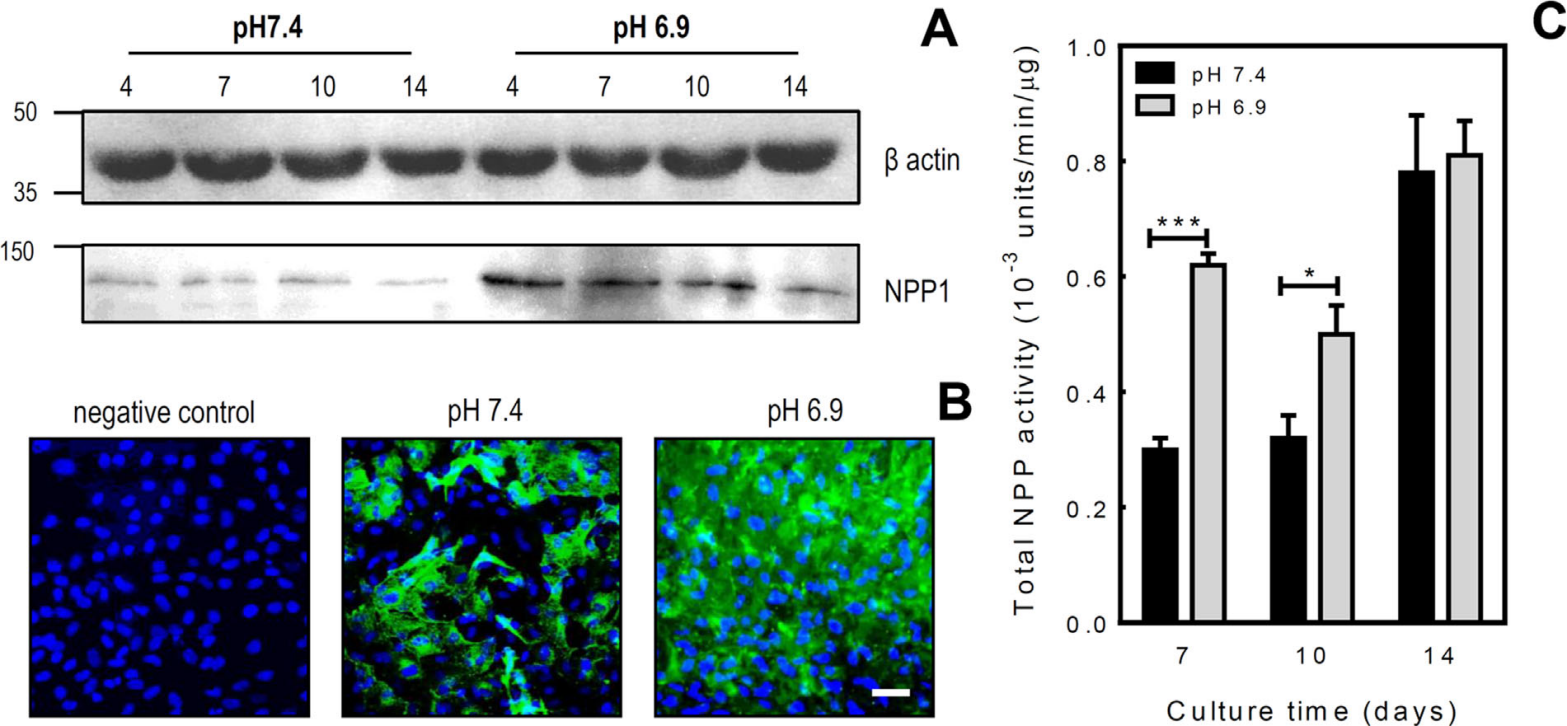
Acidosis increases NPP1 protein levels and total NPP activity in osteoblasts. A: Western blot analysis demonstrated increased NPP1 protein expression by osteoblasts grown in acid conditions. B: Immunocytochemistry demonstrated increased NPP1 expression (green) by osteoblasts cultured at pH 6.9 for 7 days compared to those cultured at pH 7.4. DAPI staining of cell nuclei is blue. Scale bar = 50 μm. C: The NPP activity was examined in osteoblast whole cell lysates after 7, 10 or 14 days of culture at pH 7.4 or 6.9. Total cellular NPP activity was doubled after 7 days at pH 6.9 and increased by ∼45% after 10 days. No differences in NPP activity were observed after 14 days in culture. Values are means ± SEM (n = 6 replicate wells), ***P < 0.001, *P < 0.05.

Western blot analysis showed that culture under acid conditions increased NPP1 expression in proliferating, differentiating and mature osteoblasts (Fig. 2A). Immunocytochemistry performed on differentiating osteoblasts (7 days at pH 7.4 or 6.9) also demonstrated increased NPP1 protein expression at pH 6.9 (Fig. 2B).

### Increased total NPP activity in osteoblasts cultured at pH 6.9

Culture at pH 6.9 also increased total cellular NPP activity; levels were doubled after 7 days at pH 6.9 and increased by ∼45% after 10 days (*P* < 0.001). Acidosis had no effect on total NPP activity after 14 days of culture (Fig. 2C).

### *Enpp1^−/−^* osteoblasts display resistance to the inhibitory effects of acidosis on bone mineralisation

**Figure 3:**
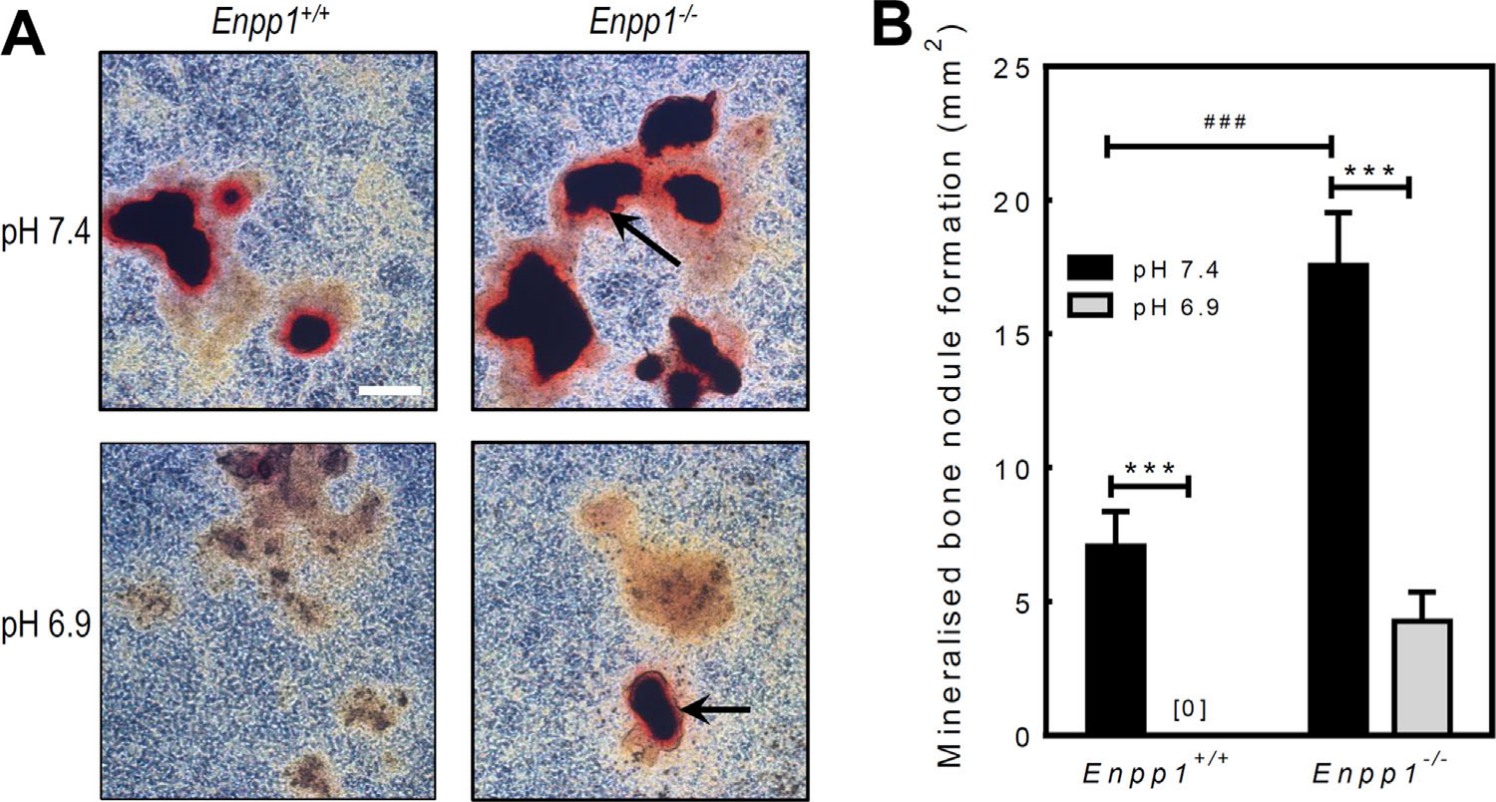
Osteoblasts from *Enpp1^−/−^* mice display resistance to the inhibitory effects of acidosis on bone mineralisation. Osteoblasts were isolated from *Enpp1^+/+^* or *Enpp1^−/−^* mice and cultured for 28 days at pH 7.4 or 6.9. A: Representative phase contrast micrographs showing alizarin red stained osteoblast cell layers from *Enpp1^+/+^*and *Enpp1^−/−^* mice at pH 7.4 and pH 6.9. *Enpp1^−/−^* osteoblasts display increased mineralisation at pH 7.3 and resistance to the inhibitory effects of acidosis on bone mineralisation. Scale bar = 500 μm. B: Bone mineralisation is increased 2.5‐fold in *Enpp1^−/−^* osteoblasts at pH7.4, compared to *Enpp1^+/+^*. Culture at pH 6.9 abolishes bone mineralisation in *Enpp1^+/+^*osteoblasts, whilst in *Enpp1^−/−^* osteoblasts mineralisation was only reduced by 75%. Values are means ± SEM (n = 6 replicate wells), ***P < 0.001.

Calvarial osteoblasts isolated from *Enpp1^+/+^* and *Enpp1^−/−^* mice were cultured for 28 days at pH 7.4 or 6.9. At a normal physiological pH 7.4, *Enpp1^−/−^* osteoblasts displayed a 2.5‐fold (*P* < 0.001) increase in bone mineralisation compared to *Enpp1^+/+^* cells (Fig. 3A and B). Reducing the pH to 6.9 resulted in a complete abolition of bone mineralisation in *Enpp1^+/+^* cells; in contrast, bone mineralisation showed only partial inhibition in *Enpp1^−/−^* osteoblasts (Fig. 3A and B).

### Acidosis does not affect ATP release by osteoblasts in vitro

**Figure 4:**
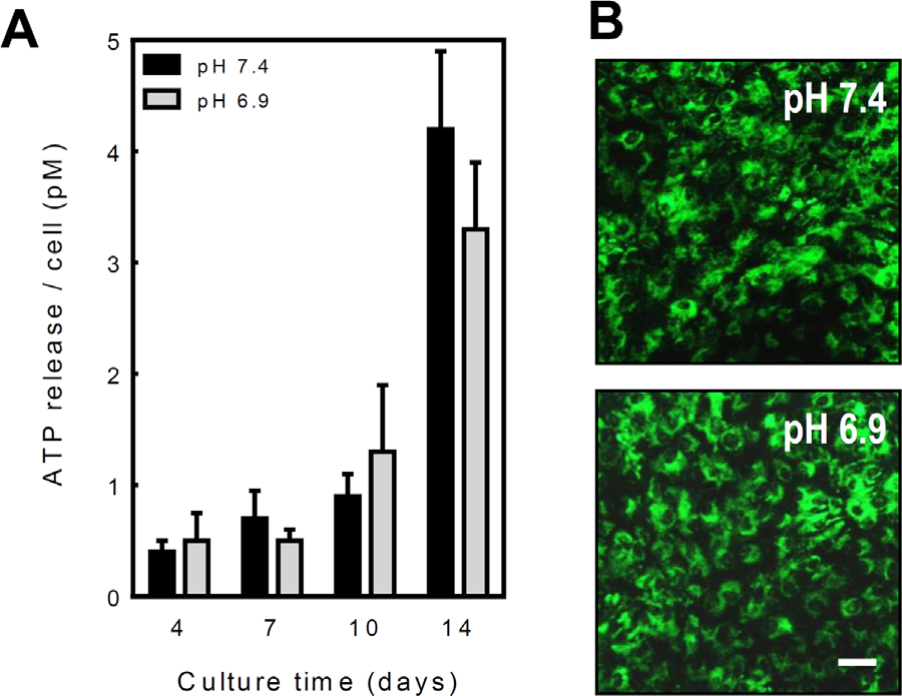
Acidosis does not affect ATP release from osteoblasts. Osteoblasts were cultured for up to 14 days at pH 7.4 or 6.9 and ATP release measured at 4, 7, 10 and 14 days. A: Basal ATP release increased with differentiation being eightfold higher in mature, bone forming osteoblasts relative to pre‐osteoblasts. Acidosis did not influence ATP release at any stage of culture. Values are means ± SEM (n = 8–10 replicate wells), ***P < 0.001. B: Osteoblasts cultured for 10 days at pH 7.4 or 6.9 showed similar levels of quinacrine staining. Scale bar = 50 μm.

ATP is a key substrate of the NPPs and is released from osteoclasts (Brandao‐Burch et al., 2012), osteoblasts (Genetos et al., 2005; Orriss et al., 2009) and osteocytes (Genetos et al., 2007; Hajjawi et al., 2014) in a controlled manner. To determine whether acidosis influenced ATP efflux and, consequently, substrate availability for NPP1, ATP release was measured in normal osteoblasts cultured at pH 7.4 or 6.9 for 4–14 days. ATP release was increased up to eightfold in mature, bone forming osteoblasts compared to pre‐osteoblasts. However, acidosis did not result in any significant changes in ATP release at any stage of the culture (Fig. 4A). For the duration of the experiment, osteoblast viability was unaffected by culture at pH 6.9 (not shown). Staining of intracellular ATP‐containing vesicles using quinacrine was similar in pH 6.9 and pH 7.4 cultures of normal osteoblasts (Fig. 4B).

## Discussion

A number of studies have now shown that acidosis exerts significant negative effects on osteoblast function (Krieger et al., 1992; Ramp et al., 1994; Sprague et al., 1994; Brandao‐Burch et al., 2005; Takeuchi et al., 2013). These effects include decreased mineralisation, reduced alkaline phosphatase (TNAP) expression and activity (Krieger et al., 1992; Brandao‐Burch et al., 2005) and also increased osteoprotegerin production (Takeuchi et al., 2013). The present investigation provides the first evidence that the pyrophosphate‐generating enzyme, NPP1, also mediates the effects of acid on osteoblasts and bone mineralisation.

Mineralisation of collagenous bone matrix is regulated by the ratio of inorganic phosphate (P_i_) to the mineralisation inhibitor, pyrophosphate (PP_i_) within the bone microenvironment. Different members of the ecto‐nucleotidase family generate either P_i_ (TNAP, NTPdases) or PP_i_ (NPPs) from extracellular nucleotides (such as ATP) released from cells. This study demonstrated that mRNAs for six members of the NTPdase family (NTPdases 1–6) are expressed by primary rat osteoblasts. In agreement with previous reports (Johnson et al., 2000; Hessle et al., 2002; Orriss et al., 2007), osteoblasts were found to also express three members of the NPP family (NPP1‐3). Marked differences in the relative levels of mRNAs for these ecto‐nucleotidases were observed in both differentiating and mature osteoblasts. NPP1 has been reported to be the most highly expressed NPP in bone (Vaingankar et al., 2004), an observation which was confirmed in our primary osteoblast cultures. We found that expression of *Enpp1* mRNA by osteoblasts was the highest of all the ecto‐nucleotidases studied here, including TNAP. In contrast, the expression of *NTPdase 1–3* was up to 10‐fold lower than TNAP, suggesting that these cell surface enzymes may be less important contributors to extracellular P_i_ levels. NTPdases 4–6 were relatively highly expressed by osteoblasts in our study; however, these enzymes are reported to be localised intracellularly (Yegutkin, 2008), suggesting that they may not contribute significantly to extracellular P_i_ levels.

Our study showed that in culture medium acidified to pH 6.9, so as to completely abolish bone mineralisation (Brandao‐Burch et al., 2005), expression of NPP1 by rat osteoblasts at both mRNA and protein levels was strikingly upregulated, as was total NPP activity. The function of *Enpp1^+/+^* mouse osteoblasts was similarly pH‐dependent, with no bone mineralisation evident in cells cultured at pH 6.9. In contrast, in cultures of *Enpp1^−/−^* osteoblasts, mineralisation was >2‐fold greater than in wildtypes at pH 7.4, and mineralisation was still evident at pH 6.9, albeit it at a significantly reduced level. This partial resistance of *Enpp1^−/−^* cells to the effects of acid provides additional evidence for the role of NPP1 in mediating the inhibitory effects of low pH on bone mineralisation. Acid upregulation of NPP1 and total NPP activity in rat osteoblasts occurred in differentiating and maturing cells at days 7–10 of culture. The lack of acid stimulation in the fully mature, bone‐forming cells (day 14) may be related in part to the negative feedback inhibition of NPP1 by extracellular ATP and its hydrolysis product, PPi (Orriss et al., 2007); our work has also shown that ATP secretion from osteoblasts is greatest when the cells are fully mature and depositing bone (Orriss et al., 2009).

This study additionally confirms the earlier reports of decreased TNAP expression and activity in acidosis (Krieger et al., 1992; Brandao‐Burch et al., 2005). The actions of TNAP are directly antagonistic to those of NPP1, and the opposing actions of these enzymes are important in maintaining mineralisation at a normal level (Johnson et al., 2000). Thus, at pH 6.9, the reduced TNAP activity combined with the increased NPP activity could shift the extracellular P_i_/PP_i_ ratio in favour of PP_i_, inhibiting bone mineralisation. Whether low pH affects the expression of other enzymes involved in regulating P_i_ availability for mineralisation, such as PHOSPHO1 (Houston et al., 2004) was not investigated here but presents a worthwhile area for future study.

Despite the decreased extracellular PP_i_ levels, the *Enpp1^−/−^* mouse model displays reduced trabecular and cortical bone in the long bones (Mackenzie et al., 2012b). In contrast, our in vitro findings show that deletion of NPP1 leads to increased levels of bone formation under normal and acid conditions. A number of factors could contribute towards the differences between the in vivo and in vitro data. Firstly, *Enpp1^−/−^* mice have a complex phenotype and thus it is possible that alterations in other tissues could indirectly influence bone mass (Mackenzie et al., 2012b); for example, *Enpp1^−/−^* animals have increased levels of serum sclerostin, a key inhibitor of bone formation (Hajjawi et al., 2014). Secondly, *Enpp1^−/−^* mice display an unusual walking gate (Sali et al., 1999), mineralisation of the joints and muscle damage (Mackenzie et al., 2012b), which combined are likely to affect the mechanical loading of bones. Finally, earlier work has suggested that the reduced bone in *Enpp1^−/−^* animals occurs because there is insufficient PP_i_ for TNAP to generate the P_i_ needed for normal bone formation (Mackenzie et al., 2012a). Osteoblasts are typically cultured in vitro with an exogenous source of P_i_ (usually β‐glycerophosphate), which provides an alternative substrate for TNAP and supply of P_i_ that is not present in vivo.

The current study used calvarial osteoblasts to examine whether acidosis regulates ecto‐nucleotidase expression and activity. Previous work demonstrated that the effects of NPP1 deletion on osteoblast function were influenced by the skeletal origin of the cells (Anderson et al., 2005); calvarial osteoblasts displayed increased mineralisation, as seen in this study, whilst bone marrow osteoblasts showed decreased mineralisation (Anderson et al., 2005). The effect of acidosis on NPP1 expression and activity in osteoblasts derived from the long bones was not examined in this investigation; however, since NPP1 expression is reported to be higher in the skull, it is possible that the effects are more pronounced in calvarial cells.

The non‐enzymatic, plasma‐membrane channel ANK is also considered to act as a PP_i_ transporter, contributing to the extracellular pool of PP_i_ (Johnson et al., 2003). We found that mRNA for ANK was highly expressed by rat osteoblasts at all stages of culture but that its expression was not significantly altered by acidosis, suggesting that this protein may not be involved in mediating the effects of acid on mineralisation.

ATP is a well‐documented inhibitor of mineralisation and its actions are thought to be mediated via both the P2 receptors and hydrolysis to produce PP_i_ (Orriss et al., 2012a, 2007). Osteoblasts release ATP constitutively under normal conditions, and this release can be enhanced by fundamental stimuli such as hypoxia (Orriss et al., 2009), fluid shear stress (Genetos et al., 2005) and mechanical loading (Rumney et al., 2012). We found in this study, however, that exposure of osteoblasts to acidic culture medium did not influence ATP release or cell viability at any stage of culture, suggesting that the inhibition of mineralisation at low pH is unlikely to be mediated by increased extracellular ATP. Instead, the decreased mineralisation is likely to involve alterations in ATP metabolism (because of differences in NPP1 and TNAP expression and activity) and, consequently, the amounts of P_i_ / PP_i_ produced.

In summary, the results presented in this study provide further evidence for the cell‐mediated actions of acid on bone mineralisation, identifying for the first time a role for NPP1 in this process. This work also adds to the growing body of evidence for the fundamental role of ecto‐nucleotidases, extracellular ATP and the P_i_ / PP_i_ ratio in the regulation of bone mineralisation.
